# Obstructive Sleep Apnea Presenting as Non-epileptic Spells: A Unique Combination

**DOI:** 10.7759/cureus.1800

**Published:** 2017-10-24

**Authors:** Prashant Natteru, Chintan Rupareliya, Xiangping Zhou, Pradeep C Bollu

**Affiliations:** 1 Department of Neurology, University of Mississippi Medical Center; 2 Department of Neurology, University of Missouri, Columbia, Missouri

**Keywords:** obstructive sleep apnea, apneic seizure, non epileptic spells, sleep related hypermotor epilepsy, pnes, nocturnal frontal lobe epilepsy

## Abstract

Obstructive sleep apnea (OSA), an increasingly prevalent sleep disorder, has been extensively studied in both clinical and scientific settings. In most cases, the diagnosis of sleep apnea is straightforward with patients having symptoms of snoring, choking or gasping for air while asleep and witnessed apneas. However, sleep apnea is known to present in some unusual ways. We present a case of a 61-year-old male, with recently diagnosed obstructive sleep apnea (currently not on continuous positive airway pressure (CPAP)) and a history of seizure-like events since the age of 18 years, who came to the epilepsy monitoring unit (EMU) for spell characterization of his frequent seizure-like episodes. A continuous video electroencephalogram (vEEG) performed in order to determine the semiology of these spells showed that all the spells were triggered by an arousal from sleep with an associated apneic event. He was started on positive airway pressure (PAP) therapy, which resulted in the gradual decline in the number as well as the severity of his seizure-like spells. Based on the observations from vEEG monitoring and the patient’s response, we concluded these seizure-like events as non-epileptic spells, triggered by apnea-related arousals in the context of OSA.

## Introduction

Obstructive sleep apnea (OSA) is a sleep disorder characterized mainly by repetitive upper airway collapse resulting in airway obstruction and nocturnal sleep fragmentation [1]. Prevalence of symptomatic OSA carries a disparity in studies performed in different countries. However, the prevalence of obstructive sleep apnea syndrome (OSAS) from the Wisconsin Sleep Cohort study in 1993 involving 602 patients is 3.3% in men and 1.2% in women [2]. Untreated OSA can result in multiple nocturnal arousals, respiratory events with oxygen desaturation, sleep fragmentation, and chronic sleep deprivation [1-2]. The consequences of untreated sleep apnea are multiple and may include cardiovascular and cerebrovascular disease, poorly controlled hypertension, and mood problems [1-2]. There is a complex and close causal relationship between sleep and seizures. Sleep deprivation has been traditionally used to trigger seizures in the setting of epilepsy during routine diagnostic testing. Frontal lobe seizures are more common during sleep; nonetheless, the most common nocturnal seizures is still a temporal lobe seizure [3]. Our patient had multiple events mimicking generalized tonic-clonic seizure (GTCS), also known as "grand mal", from nocturnal arousals in the background of his undiagnosed OSA.

## Case presentation

A 61-year-old man with a past medical history of cerebral palsy (CP), obstructive sleep apnea, and intractable epilepsy visited our neurology clinic with chief complaints of progressive worsening of his seizure events with the frequency of eight to nine times every night. His seizure-like spells were happening both in nighttime sleep and during the daytime naps. He was first diagnosed with seizures at the age of 18 years and had been on multiple anti-epileptic drugs including zonisamide, phenytoin, lacosamide, and clonazepam for the past 35 years.

The semiology of his typical spell consisted of right lower extremity movements that would then generalize to involve all four limbs. The patient reported an aura of "feeling tense" before the spell. The motor activity would last anywhere from thirty seconds to one minute. However, the patient was conscious and was able to call out to the family for help. His spells were not associated with tongue biting and bowel or bladder incontinence. He reported feeling tired postictally but rarely has any confusion after the spell.

**Figure 1:**
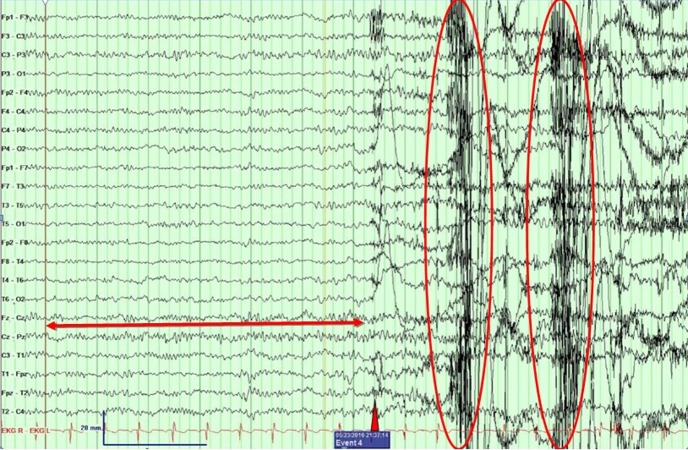
Pre-event epoch An epoch showing baseline normal EEG at sleep on the left (red two-headed arrow), a point of arousal (red triangle), and generalized movement discharges (red circles). No epileptiform interictal discharges or signs of lateralization are seen.

**Figure 2:**
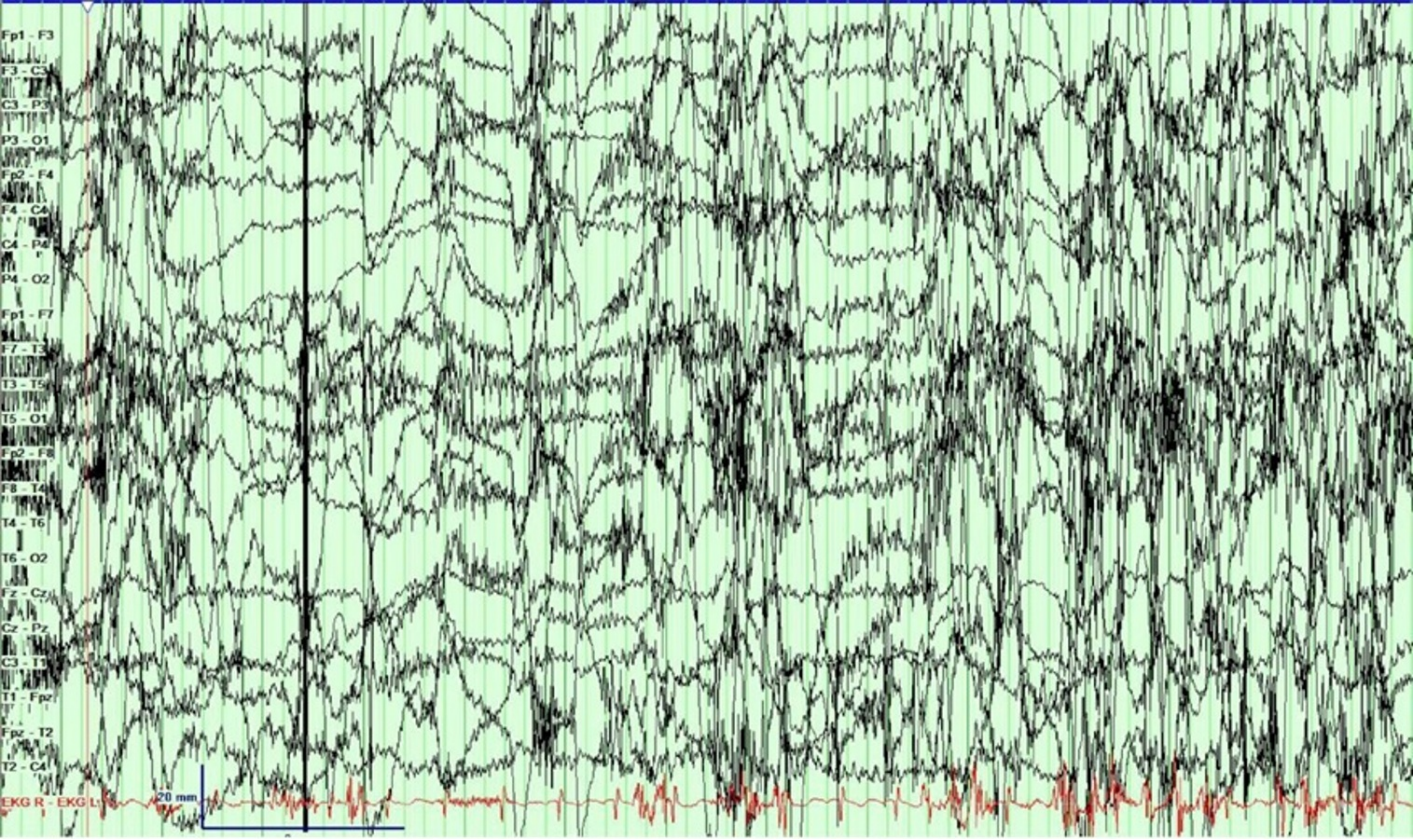
An epoch during continuous seizure Electroencephalogram (EEG) during an ongoing spell. The patient seems to have woken up as seen by eye blinks intermixed with movement artifacts. However, no epileptiform discharges are seen.

**Figure 3:**
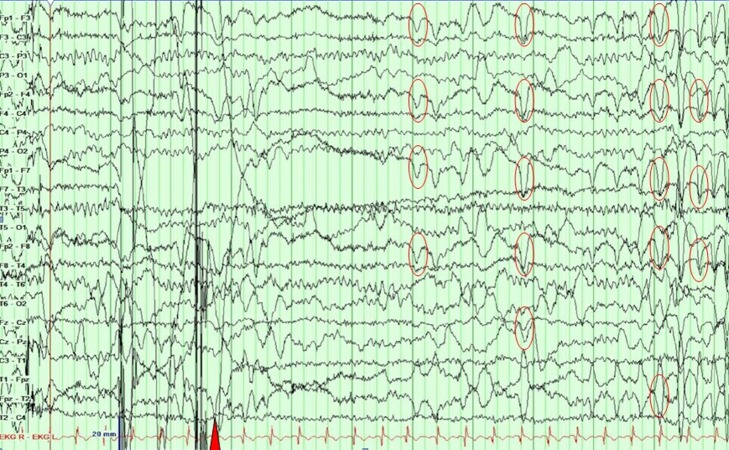
An epoch showing an EEG returning to baseline (red triangle), and the patient is awake, seen as eye blinks (red circles) A clinical seizure ended at the point marked by the red triangle.

He was admitted to the epilepsy monitoring unit (EMU) for characterization of the spells. His clinical examination and labs were unremarkable with both phenytoin and zonisamide levels being in the subtherapeutic range at the time of admission. During his stay in the EMU, a 16-channel video electroencephalogram (vEEG) monitoring was performed to characterize these spells. All of his antiepileptic drugs (AED) were held for the recording. On the first night he had five spells, and all of them occurred during sleep and manifested as arousals with an apneic event. None of the spells had any epileptiform correlate on the vEEG, as demonstrated in Figure 1. Figure 2 shows high amplitude delta activity intermixed with movement artifacts. Figure 3 marks the end of an event with EEG findings returning to the baseline.

Based on these findings, we concluded that these non-epileptic spells were, in fact, obstructive apneas, and the patient was started on CPAP therapy. Over the course of his remaining stay in the EMU, there was a significant decline in the number of spells after starting CPAP. There were no abnormal ictal and/or interictal discharges on the EEG. He was discharged with education about non-epileptic seizures and the prognosis of this condition. During the follow-up appointment in the clinic, the patient reported complete resolution of his spells. A telephonic consent was obtained from the patient by one of the authors of the manuscript to report his case for a journal publication.

## Discussion

Obstructive sleep apnea (OSA) is a sleep disorder that causes breathing interruptions lasting from a few seconds to a minute [1]. The pathogenic link between sleep apnea and seizures has been researched extensively and is thought to be due to increased neuronal excitability and synchronized discharge of neurons [2]. One of the many explanations of seizure aggravation in epilepsy due to obstructive sleep apnea are intermittent hypoxia (IH) leading to potential regional brain injury and sleep fragmentation (SF). Both IH and SF, possibly have an additive effect towards brain damage [3]. Sleep apnea is commonly associated with refractory epilepsy, and there is evidence showing that there is a benefit with continuous positive airway pressure (CPAP) therapy for refractory epilepsy patients with obstructive sleep apnea [4].

In our patient, the presentation of seizures occurring mainly during sleep (nocturnal and daytime naps) raised a suspicion of temporal lobe epilepsy or nocturnal frontal lobe epilepsy (NFLE). About 70% of the patients with sleep-related temporal lobe epilepsy wake up from sleep with an aura, progressing to a complex partial seizure and secondary generalization tonic-clonic seizures. The seizures in nocturnal temporal lobe epilepsy (NTLE), a subtype of medically refractory temporal epilepsy, almost occur exclusively at night [5].

Seizures in NFLE most commonly occur during non-rapid eye movement (NREM) sleep and are often preceded by a sudden arousal or a distinct aura. These patients may also complain of disturbed sleep and daytime tiredness. Seizures in NFLE vary from stereotyped arousals to complex hypermotor behaviours (rocking body movements with kicking or cycling of limbs) [6]. Temporal lobe seizures when generalized can also present as hypermotor seizures.

In our patient, after extensive five-day vEEG monitoring, all the spells were found to be non-epileptic and were noted to be associated with an arousal from sleep due to an apneic event due to obstructive sleep apnea. These spells became less frequent with the initiation of CPAP therapy. The patient eventually reported complete freedom from his spells.

Commonly misdiagnosed as epilepsy, patients with non-epileptic spells are often treated with antiepileptic drugs for years before an actual diagnosis is made. Non-epileptic seizures are not uncommon in clinical practice, with most patients having associated psychiatric comorbidities. Some patients can have a combination of epileptic seizures and non-epileptic spells. A study by Benbadis, et al. showed that psychogenic non-epileptic epilepsy seizure (PNES) was the most frequent non-epileptic condition presenting at epilepsy centers, more common than physiological non-epileptic conditions [7].

Video-EEG monitoring is the gold standard to differentiate non-epileptic seizures from true epileptic seizures. Certain features that increase the likelihood of non-epileptic seizures include gradual onset or termination, stop-and-go movements, out-of-phase activity, opisthotonic posturing, and awareness during the spell. Pelvic thrusting, however, is seen in both non-epileptic seizures and frontal lobe epilepsy [8].

Appropriate education about the nature of the non-epileptic seizure and adequate management of associated psychiatric disorder is crucial in treating PNES. In one study, when the diagnosis of PNES was conveyed properly and accepted by the patients, up to 30% of the patients enjoyed remission of PNES, often not needing any further intervention [9-10].

## Conclusions

Obstructive sleep apnea is a sleep-related breathing disorder with a variety of manifestations. In our case, the respiratory events associated with the patient’s sleep apnea were mistaken for epileptic seizure and were being treated with antiepileptic medications. A video-EEG recording was able to finally prove the nature of the spells, and treatment of the sleep apnea with CPAP alleviated the patient’s problem. Our case emphasizes the need to understand that not all nocturnal spells are seizures and that proper identification of the nature of the spells is important to address them appropriately.
